# A nonchlorinated solvent-processed polymer semiconductor for high-performance ambipolar transistors

**DOI:** 10.1093/nsr/nwab145

**Published:** 2021-08-14

**Authors:** Jie Yang, Yaqian Jiang, Zhiyuan Zhao, Xueli Yang, Zheye Zhang, Jinyang Chen, Junyu Li, Wei Shi, Shuai Wang, Yunlong Guo, Yunqi Liu

**Affiliations:** Beijing National Laboratory for Molecular Sciences, Key Laboratory of Organic Solids, Institute of Chemistry, Chinese Academy of Sciences, Beijing100190, China; School of Chemistry and Chemical Engineering, Huazhong University of Science and Technology, Wuhan430074, China; Beijing National Laboratory for Molecular Sciences, Key Laboratory of Organic Solids, Institute of Chemistry, Chinese Academy of Sciences, Beijing100190, China; Beijing National Laboratory for Molecular Sciences, Key Laboratory of Organic Solids, Institute of Chemistry, Chinese Academy of Sciences, Beijing100190, China; Beijing National Laboratory for Molecular Sciences, Key Laboratory of Organic Solids, Institute of Chemistry, Chinese Academy of Sciences, Beijing100190, China; School of Chemistry and Chemical Engineering, Huazhong University of Science and Technology, Wuhan430074, China; Beijing National Laboratory for Molecular Sciences, Key Laboratory of Organic Solids, Institute of Chemistry, Chinese Academy of Sciences, Beijing100190, China; Beijing National Laboratory for Molecular Sciences, Key Laboratory of Organic Solids, Institute of Chemistry, Chinese Academy of Sciences, Beijing100190, China; Beijing National Laboratory for Molecular Sciences, Key Laboratory of Organic Solids, Institute of Chemistry, Chinese Academy of Sciences, Beijing100190, China; School of Chemistry and Chemical Engineering, Huazhong University of Science and Technology, Wuhan430074, China; Beijing National Laboratory for Molecular Sciences, Key Laboratory of Organic Solids, Institute of Chemistry, Chinese Academy of Sciences, Beijing100190, China; Beijing National Laboratory for Molecular Sciences, Key Laboratory of Organic Solids, Institute of Chemistry, Chinese Academy of Sciences, Beijing100190, China

**Keywords:** nonchlorinated solvent, high performance, ambipolar transistor, alignment

## Abstract

Ambipolar polymer semiconductors are potentially serviceable for logic circuits, light-emitting field-effect transistors (LFETs) and polymer solar cells (PSCs). Although several high-performance ambipolar polymers have been developed, their optoelectronic devices are generally processed from toxic chlorinated solvents. To achieve the commercial applications of organic FETs (OFETs), the polymers should be processed from nonchlorinated solvents, instead of chlorinated solvents. However, most conjugated polymers show poor solubility in nonchlorinated solvents. It is of great importance to develop ambipolar polymers that can be processed from nonchlorinated solvents. Here, we develop a nonchlorinated solvent processed polymer named poly[7-fluoro-N, N^′^-di(4-decyltetradecyl)-7^′^-azaisoindigo-6^′^,6^″^-(thieno[3,2-b]thiophene-2,5-diyl)-7‴-fluoro-N^″^, N‴-di(4-decyltetradecyl)-7^″^-azaisoindigo-6,6‴-([2,2^″^-bithiophene]-5,5^″^-diyl)] (PITTI-BT) by designing a monomer with a large molar mass. The polymer displays good solubility in p-xylene (PX). Well-aligned films of PITTI-BT are achieved by an off-center spin-coating (SC) method. Based on the high-quality films, the OFETs fabricated from PX solution achieve record ambipolar performance with hole and electron mobilities of 3.06 and 2.81 cm^2^ V^−1^ s^−1^, respectively. The combination of nonchlorinated solvents and good alignment process offers an effective and eco-friendly approach to obtain high-performance ambipolar transistors.

## Teaser

A nonchlorinated solvent-processed isoindigo-based polymer is developed, which can form well-aligned films and achieve outstanding ambipolar performance with hole and electron mobilities of 3.06 and 2.81 cm^2^ V^−1^ s^−1^ in organic field-effect transistors.

## INTRODUCTION

Solution-processable semiconducting polymers have wide applications in OFETs, PSCs, organic thermoelectrics (OTEs) and organic electrochemical transistors (OECTs) [1–12]. To date, various p-type polymers have been achieved based on well-known building blocks such as diketopyrrolopyrrole (DPP) and isoindigo (IID) [13–15]. However, the advances of ambipolar polymers lag behind those of p-type counterparts. There remains a need to develop ambipolar polymers because of their potential applications in flexible optoelectronic devices such as logic circuits, LFETs and PSCs [6,16–19]. Several strategies have been explored to promote ambipolar transport by introducing electron-withdrawing groups (F, Cl, N, CN, etc.) or acceptor dimers into the polymer backbones [20–25]. Recently, several polymers demonstrated excellent ambipolar transport with both hole and electron mobilities (*μ*_h_ and *μ*_e_) > 2 cm^2^ V^−1^ s^−1^ [25–29]. However, almost all high-mobility ambipolar devices are processed from hazardous chlorinated solvents, such as chlorobenzene (CB) or o-dichlorobenzene (ODCB) [4]. These solvents do not exist in nature and require to be artificial synthesized from aromatic compounds. Moreover, chlorinated solvents are harmful to people and may cause environmental pollution, which will block future commercial applications of OFETs. By comparison, nonchlorinated solvents are better candidates for OFET fabrication because they are more eco-friendly and available from petroleum. However, most polymers show poor solubility in nonchlorinated solvents. The electron mobilities of nonchlorinated solvent-processed ambipolar polymers are still below 1 cm^2^ V^−1^ s^−1^ (Supplementary Table S1). Therefore, it is an important research target to explore nonchlorinated solvent-processed ambipolar polymers.

To achieve nonchlorinated solvent-processed polymers, three synthetic strategies are adopted: (1) enriching the proportion of alkyl chains in polymers, (2) introducing asymmetric monomers and (3) using random copolymerization [30–34]. However, most of polymers synthesized using such strategies achieved only p-type charge transport. In addition to material design, solution-processed techniques are other important factors that can influence mobilities. The possibility of improving polymer mobilities by aligning polymer molecules has been demonstrated by several research groups [35–40]. For instance, our group recently reported a well-aligned polymer using a bar-coating method, which achieved remarkable performance with *μ*_h_ and *μ*_e_ of 5.5 and 4.5 cm^2^ V^−1^ s^−1^, respectively [40]. Nevertheless, it should be noted that almost all of the aligned high-mobility polymers reported were processed from toxic chlorinated solvents. Aligned ambipolar nonchlorinated solvent-processed polymers were rarely reported.

Isoindigo is a well-known building block from which to construct high-mobility polymers. Isoindigo-based polymers have distinctive features including facile material synthesis, good coplanarity and high crystallinity [14]. However, most isoindigo-based polymers are hardly soluble in nonchlorinated solvents, which might be attributed to their large molecular weights or strong intermolecular interaction [20,24,27,41]. Here, we developed an isoindigo-based nonchlorinated solvent-processed polymer (PITTI-BT) by designing a monomer with a large molar mass. PITTI-BT possessed a low number-average molecular weight (*M*_n_) of 18.3 kDa and was soluble in PX. To date, only several material systems such as cyclopentadithiophene (CDT) and naphthalenediimide (NDI) have achieved good alignment [35,36,42]. The studies of aligned isoindigo-based polymers are very limited [40]. We attempted to explore the possibility of polymer alignment based on PITTI-BT using an off-center SC method. As evidenced by atomic force microscopy (AFM) and two-dimensional grazing incidence wide-angle X-ray scattering (2D-GIWAXS), off-center spin-coated films from PX solution achieved well-aligned edge-on alignment, which was favorable for charge transport. Based on high-quality films, the OFETs showed remarkable ambipolar performance with a *μ*_h_/*μ*_e_ of 3.06/2.81 cm^2^ V^−1^ s^−1^, reaching the highest values reported for nonchlorinated solvent-processed ambipolar devices. For comparison, OFETs were also fabricated from ODCB solution and achieved *μ*_h_ and *μ*_e_ of 4.72 and 4.11 cm^2^ V^−1^ s^−1^, respectively.

## RESULTS AND DISCUSSION

### Synthesis, optical and electrochemical properties

**Scheme 1.**
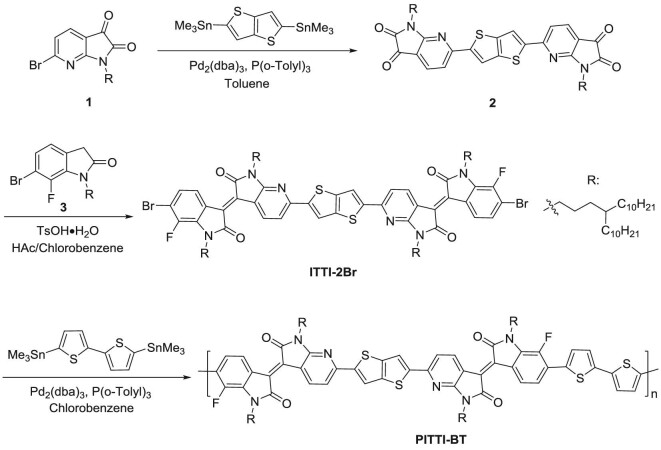
The route to PITTI-BT.

**Figure 1.**
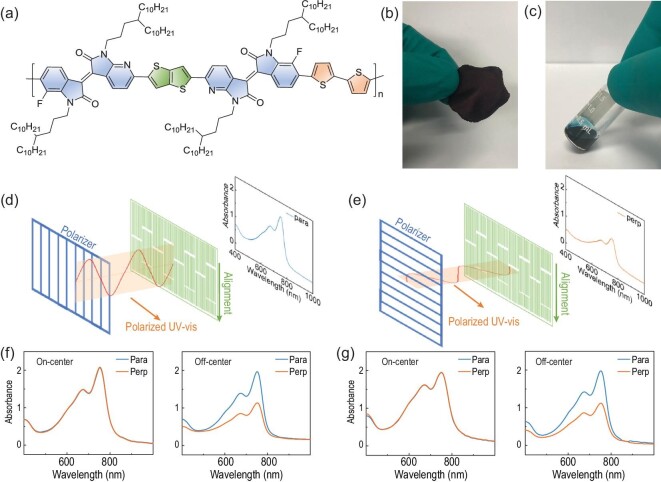
(a) Molecular structure of PITTI-BT. (b) Photograph of PITTI-BT film. (c) PX solution of PITTI-BT. (d, e) Schematic of polarized UV-vis tests of the off-center spin-coated films. The light polarization direction is (d) parallel or (e) perpendicular to the film aligned direction. The same film areas were selected for tests. Polarized UV-vis absorption spectra of the on-center or off-center spin-coated films prepared from (f) PX or (g) ODCB solution.

Scheme 1 presents the synthetic route to PITTI-BT. First, a Stille coupling reaction between compound 1 and 2,5-bis(trimethylstannyl)thieno[3,2-b]thiophene afforded compound 2. The monomer 6,6‴-dibromo-7-fluoro-N,N^′^-di(4-decyltetradecyl)-7^′^-azaisoindigo-6^′^,6^″^-(thieno[3,2-b]thiophene-2,5-diyl)-7‴-fluoro-N^″^, N‴-di(4-decyltetradecyl)-7^″^-azaisoindigo (ITTI-2Br) was obtained by a condensation reaction between compound 2 and compound 3. Stille coupling polymerization between ITTI-2Br and 5,5^″^-bis(trimethylstannyl)-2,2^″^-bithiophene gave the polymer PITTI-BT (Supplementary Fig. 1). The monomer has a large molar mass of 2203 g/mol, which may slow down the polymerization reaction rate and decrease the polymer molecular weight [43]. PITTI-BT showed a low *M*_n_ of 18.3 kDa and was highly soluble in common solvents. PITTI-BT showed good solubility in CB (∼20 mg/mL), ODCB (∼25 mg/mL) and PX (∼20 mg/mL) (Supplementary Fig. 2 and Fig. 1c). A thermogravimetric analysis (TGA) curve indicated that PITTI-BT showed good thermal stability with a decomposition temperature > 405°C (Supplementary Fig. 4). Density functional theory (DFT) calculations were carried out to investigate the optimized geometry of PITTI-BT (Supplementary Figs 5 and 6). The dihedral angle between the two indolone subunits was 8.8°.

The UV−vis absorption spectra of PITTI-BT were recorded both in solution and in thin film. PITTI-BT showed an optical bandgap (E_g_^opt^) of 1.52 eV calculated from the film absorption onset (Supplementary Fig. 7). Temperature-dependent UV–vis spectroscopy was further performed to investigate the aggregation behavior of PITTI-BT in dilute PX and ODCB solutions (0.025 mg/mL). PITTI-BT showed an intramolecular charge transfer (ICT) transition peak at 671 nm with an obvious shoulder peak at 745 nm in PX at 30°C (Supplementary Fig. 8). As the temperature increased, negligible change was observed for the maximum absorption peak at 671 nm. However, the shoulder peak at 745 nm gradually became weaker and blue shifted with increasing temperature. As shown in Supplementary Fig. 8, a similar trend was observed for absorption spectra of PITTI-BT in ODCB. These results demonstrated that PITTI-BT formed aggregation both in PX and ODCB solutions. Cyclic voltammetry (CV) of PITTI-BT was evaluated (Supplementary Fig. 9). The highest occupied molecular orbital (HOMO) energy level of PITTI-BT was −5.71 eV, which was calculated from the onset of the oxidation peak of the CV curve using the equation E = −(E_onset_ + 4.40 eV). As the reduction peak of PITTI-BT was weak, the lowest unoccupied molecular orbital (LUMO) energy level (−4.19 eV) was calculated using the equation: E_g_^opt^ = LUMO–HOMO [21,44].

### Film morphology and alignment

**Figure 2.**
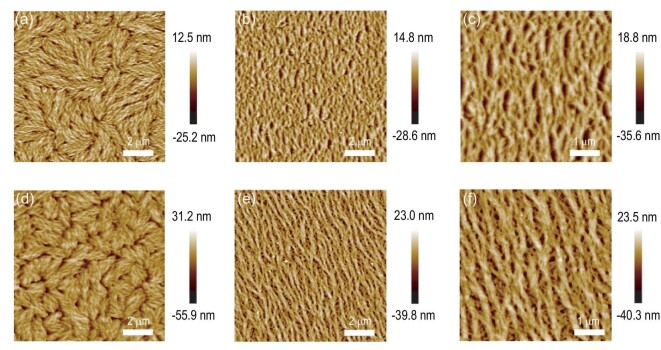
AFM height images of the annealed polymer films prepared by (a, d) on-center and (b, c, e, f) off-center SC methods from (a–c) PX or (d–f) ODCB solutions.

Polymer films of PITTI-BT were prepared by on-center and off-center SC methods from PX or ODCB solutions. Supplementary Fig. 10 displays the schematic illustration of the off-center SC method. In the off-center SC process, the fast rotation of the spin coater induces a radial force, which can drive the polymer to align along the radial direction [45,46]. AFM was performed to investigate the surface morphology of the films. Annealed films were spin-coated from 5 mg/mL solution in PX for AFM measurements. Figure 2a–c shows the AFM images of films prepared by on-center and off-center SC methods from PX. As shown in Fig. 2a, the on-center spin-coated films showed randomly granular domains. In comparison, the off-center spin-coated films displayed long-range alignment along the radial coating direction (Fig. 2b).

Figure 2d–f displays AFM images of annealed films prepared from 8 mg/mL ODCB solution. The on-center spin-coated films prepared from ODCB exhibited similar surface morphology relative to those prepared from PX (Fig. 2d). In comparison, the off-center spin-coated films displayed highly ordered microstructures with long-range alignment along the radial coating direction in a scale of 10 × 10 μm (Fig. 2e). From the magnified AFM image (5 × 5 μm), we could observe that the polymer chains assembled into fibrillar bundles with diameters of 200−300 nm (Fig. 2f). Therefore, we successfully achieved well-aligned films from both PX and ODCB solutions, which are rarely reported in the literature [30]. These well-aligned films could be effective for charge transport. To further confirm the presence of an anisotropic alignment in PITTI-BT-based films, we measured polarized UV−vis absorption. Figure 1f and g shows the polarized UV−vis spectra of the on-center or off-center spin-coated films from PX and ODCB solution. The off-center spin-coated films displayed strong dependence on the absorption intensity according to the parallel and perpendicular sample orientation, demonstrating the presence of polymer alignment in the films. In comparison, the on-center spin-coated films did not exhibit any dependence on polarized light. The maximum absorption peak dichroic ratio (*R*) can be calculated using the equation *R *= *I*_//_/*I*_⊥_, where *I*_/__/_ or *I*_⊥_ represents peak absorbance with the film aligned direction parallel or perpendicular to the light polarization direction, respectively (Fig. 1d and e) [47–49]. The *R* of the films prepared from PX or ODCB was 2.1 (at *λ* = 674 nm) or 1.8 (at *λ *=* *673 nm), respectively.

### Film crystallinity

**Figure 3.**
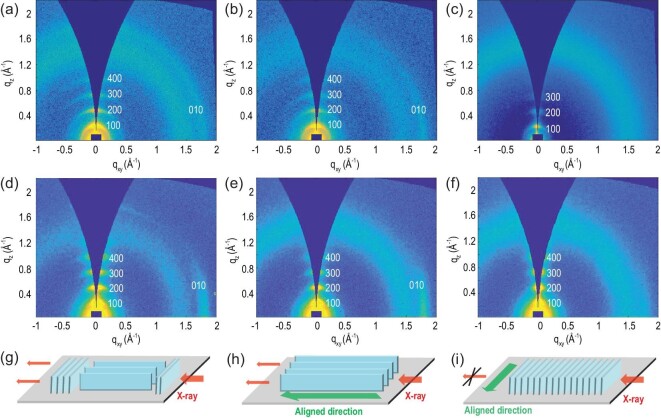
2D-GIWAXS of polymer films prepared from PX solution by (a) on-center SC, (b) off-center SC (parallel) and (c) off-center SC (perpendicular) methods. 2D-GIWAXS of polymer films prepared from ODCB solution by (d) on-center SC, (e) off-center SC (parallel) and (f) off-center SC (perpendicular) methods. (g) Schematic of molecular packing of on-center SC films. Schematics of film molecular packing are shown with the incident in-plane X-rays (h) parallel or (i) perpendicular to the film aligned direction.

2D-GIWAXS was conducted to explore the molecular alignment of PITTI-BT. Polymer films were deposited by on-center and off-center SC methods from PX solution (Fig. 3a–c). The detailed crystallographic parameters extracted from Supplementary Fig. 11 are listed in Supplementary Table S3. As shown in Fig. 3a, the film showed (*h*00) diffraction peaks up to fourth order along the out-of-plane (*q*_z_) direction, indicating the formation of well-ordered molecular packing. A (100) diffraction peak was found at *q*_z_ = 0.257 Å^−1^, corresponding to a distance of 24.4 Å, which was a typical lamella packing distance of the alkyl chains (Supplementary Fig. 11). In the in-plane direction, there was a (010) diffraction peak at *q*_xy_ = 1.80 Å^−1^, corresponding to a distance of 3.49 Å, which was a typical π−π stacking distance. Figure 3b displays the GIWAXS image of the aligned film with the incident X-rays parallel to the aligned direction (Fig. 3h). The scattering pattern of Fig. 3b was similar to that of Fig. 3a, with close lamella and π−π stacking distances. The alkyl chain stacking peaks mainly existed in the out-of-plane direction and the π−π stacking peak existed in the in-plane direction (Supplementary Fig. 11b), which indicated an edge-on dominant orientation. This edge-on orientation was favorable for charge transport [3,40]. By rotating the sample 90°, GIWAXS was measured with X-rays perpendicular to the aligned direction (Fig. 3i). As shown in Fig. 3c, an obvious difference was found for the π−π stacking peaks. The π−π stacking diffraction signals were relatively strong in the parallel condition (Fig. 3b) and nearly negligible in the perpendicular condition (Fig. 3c).

For comparison, GIWAXS of the films prepared from ODCB was also conducted (Fig. 3d–f). The films prepared from ODCB exhibited similar scattering patterns relative to those prepared from PX. All of the films showed fourth (*h*00) diffraction peaks. The diffraction pattern of Fig. 3d was similar to that of Fig. 3a, with close π−π stacking distances. In addition, the π−π stacking diffraction signals were observed with the incident X-rays parallel to the film aligned direction (Fig. 3e) and rather weak in the perpendicular condition (Fig. 3f).

### Organic field-effect transistor devices

Top-gate bottom-contact (TGBC) FETs with a device configuration of glass/Au/semiconductor/PMMA/Al were fabricated to investigate the charge transport properties of PITTI-BT (Supplementary Fig. 13). The Au/Cr (25 nm/5 nm) source and drain electrodes were patterned using a photolithography process with a channel width and length of 1400 and 40 μm, respectively. A polymer semiconducting layer was then spin-coated from PX solution. Subsequently, PMMA was used as a dielectric layer and thermally deposited aluminum served as a gate. The fabrication procedures of FET devices are provided in the Supplementary data.

**Figure 4.**
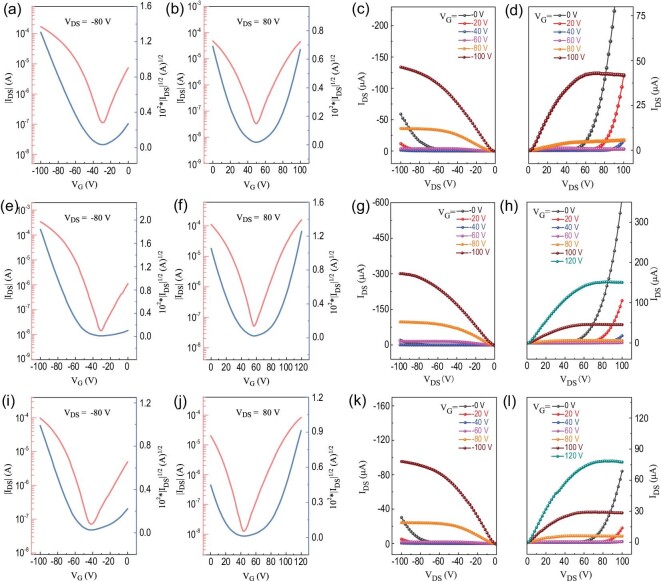
Transfer and output characteristics of OFETs prepared by (a–d) on-center and (e–l) off-center SC methods from PX solution. The transistor channels are (e–h) parallel or (i–l) perpendicular to the film aligned direction.

**Figure 5.**
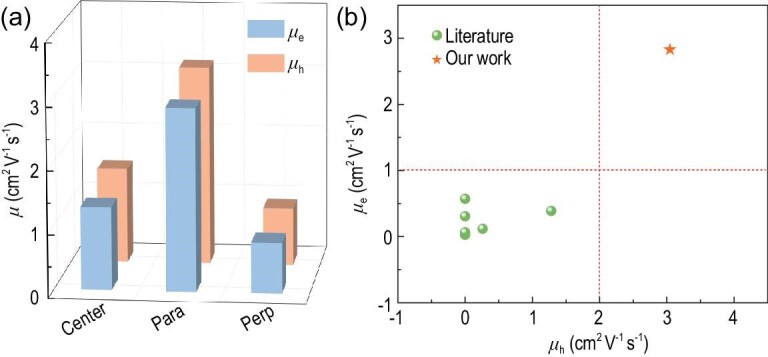
(a) The maximum mobility distribution of OFETs fabricated by on-center and off-center SC methods from PX solution. (b) Plot of *μ*_e_ versus *μ*_h_ for ambipolar or n-type OFETs fabricated from nonchlorinated solvents. Source references for the data points are provided in Supplementary Table S1.

Figure 4 shows the transfer and output curves of OFETs fabricated by on-center and off-center SC methods from PX solution. All the devices showed ambipolar transport properties with typical V-shaped transfer curves. Saturation mobilities were extracted from the slope of I_DS_^1/^^2^ vs V_G_ curves. Supplementary Table S5 provides the carrier mobilities, together with the corresponding threshold voltages and on/off current ratios. The average *μ*_h_ and *μ*_e_ of OFETs prepared by an on-center SC method were 1.06 and 0.95 cm^2^ V^−1^ s^−1^, respectively. We next examined the performance of devices that were fabricated by an off-center SC method. In the off-center SC process, the devices were located from the rotational center with a distance of 2 cm (Supplementary Fig. 10). We defined two orientations for the OFET devices: parallel (para,//), where the transistor channels were parallel to the film aligned direction, and perpendicular (perp, ⊥), where the channels were perpendicular to the aligned direction (Supplementary Fig. 14). The parallel OFETs showed remarkable ambipolar performance with an average *μ*_h,__/__/_/*μ*_e,__/__/_ of 2.31/1.87 cm^2^ V^−1^ s^−1^ and a maximum *μ*_h,__/__/_/*μ*_e,__/__/_ of 3.06/2.81 cm^2^ V^−1^ s^−1^, reaching the highest values reported for nonchlorinated solvent-processed ambipolar or n-type polymers (Fig. 5). The mobilities of parallel OFETs were about twice those of the on-center spin-coated devices. The perpendicular OFETs showed moderate performance with an average *μ*_h,__⊥_/*μ*_e,__⊥_ of 0.69/0.59 cm^2^ V^−1^ s^−1^ and a maximum *μ*_h,__⊥_/*μ*_e,__⊥_ of 0.91/0.80 cm^2^ V^−1^ s^−1^.

For comparison, we selected the common chlorinated solvent (ODCB) to fabricate OFETs and investigate their performance (Supplementary Fig. 15). As shown in Supplementary Table S5, the OFETs prepared by an on-center SC method exhibited average *μ*_h_ and *μ*_e_ of 1.57 and 1.20 cm^2^ V^−1^ s^−1^, respectively. Supplementary Fig. 15e−l provides the transfer and output curves of OFETs fabricated by an off-center SC method. For the parallel OFETs, we achieved an average *μ*_h,__/__/_/*μ*_e,__/__/_ of 4.14/3.53 cm^2^ V^−1^ s^−1^, with a maximum *μ*_h,__/__/_/*μ*_e,__/__/_ of 4.72/4.11 cm^2^ V^−1^ s^−1^, which were among the highest values for ambipolar polymers [4]. For the perpendicular OFETs, we obtained an average *μ*_h,__⊥_/*μ*_e,__⊥_ of 0.87/0.67 cm^2^ V^−1^ s^−1^ and a maximum *μ*_h,__⊥_/*μ*_e,__⊥_ of 1.21/1.03 cm^2^ V^−1^ s^−1^. The stability of OFET devices was investigated. The devices remained stable during 15 days when stored in a nitrogen glovebox (Supplementary Figs 16a and 17). The electron mobilities showed degradation when stored in air (Supplementary Figs 16b and 18). The inferior device stability of n-type transport in air might be attributed to generation of electron traps by water or oxygen in air during the device storage [50–52]. The air stability of ambipolar or n-type OFET devices is a well-known challenge in this field, which is also reported in the literature [27,53–56].

We noted that the parallel OFETs displayed obviously enhanced performance compared to the on-center spin-coated devices prepared from PX and ODCB solutions (Supplementary Table S5). This phenomenon was consistent with above AFM and GIWAXS data. In on-center spin-coated devices, the polymer backbone orientation was random, and thus charge transport was isotropic with respect to transistor channel (Supplementary Fig. 14a). In the parallel OFETs, the long-range alignment of domains and polymer backbone were parallel to the direction of charge transport, which was favorable to improve the mobilities (Supplementary Fig. 14b) [47,57–59].

## CONCLUSION

In conclusion, a nonchlorinated solvent-processed isoindigo-based polymer (PITTI-BT) was developed. This polymer showed high crystallinity and strong intermolecular interaction. Using an off-center SC method, we successfully achieved long-range aligned films from PX solution. As a result, the parallel OFETs prepared by an off-center SC method exhibited mobility twice that of devices prepared by an on-center SC method. The parallel OFETs achieved record ambipolar performance with a *μ*_h_/*μ*_e_ of 3.06/2.81 cm^2^ V^−1^ s^−1^. The combination of nonchlorinated solvents and good alignment achieves high performance ambipolar transistors, which meets the requirements of future commercial applications of OFETs.

## METHODS

### Synthesis of PITTI-BT

ITTI-2Br (100.0 mg, 0.0454 mmol), 5,5^′^-bis(trimethylstannyl)-2,2^′^-bithiophene (22.3 mg, 0.0454 mmol), catalytic tris(dibenzylideneacetone)dipalladium(0) (Pd_2_(dba)_3_, 1.3 mg), tri(o-tolyl)phosphine (P(*o*-tol)_3_, 3.5 mg) and chlorobenzene (4 mL) were added to a Schlenk tube. The tube was loaded with argon through a freeze-pump-thaw cycle in liquid nitrogen for three times. Then the reaction solution was stirred and heated at 120°C for 36 hours. The cooled reaction solution was poured into 100 mL methanol (containing 5 mL HCl) solution and stirred for 3 hours. The crude polymer was collected and purified via successive Soxhlet extraction. The first extraction solvent was methanol, then changed to acetone, hexane and chloroform. The purified polymer was finally extracted in chloroform, which was concentrated and precipitated into methanol (100 mL) to give the polymer (95 mg, 94.8%). GPC: *M*n = 18.3 kDa, *M*w = 69.5 kDa, PDI = 3.80. Anal. calcd for C_140_H_210_F_2_N_6_O_4_S_4_: C 76.17, H 9.59, N 3.81; found: C 75.87, H 9.48, N 3.78.

## Supplementary Material

nwab145_Supplemental_File
