# Supplementary material for: A nonchlorinated solvent-processed polymer semiconductor for high-performance ambipolar transistors
Source: Natl Sci Rev. 2021 Aug 14;9(4):nwab145. doi: 10.1093/nsr/nwab145 (PMC9031015; doi:10.1093/nsr/nwab145)
Supplement: nwab145_Supplemental_File [file nwab145_supplemental_file.doc]

## Supplementary material

**A nonchlorinated solvent-processed polymer semiconductor for high-performance ambipolar transistors**

Jie Yang^1,2,†^, Yaqian Jiang^1,†^, Zhiyuan Zhao^1,†^, Xueli Yang^1,†^, Zheye Zhang^2^, Jinyang Chen^1^, Junyu Li^1^, Wei Shi^1^, Shuai Wang^2^, Yunlong Guo^1^,* and Yunqi Liu^1^,*

^1^Beijing National Laboratory for Molecular Sciences, Key Laboratory of Organic Solids, Institute of Chemistry Chinese Academy of Sciences, Beijing 100190, China
^2^School of Chemistry and Chemical Engineering, Huazhong University of Science and Technology, Wuhan 430074, China

**∗Corresponding author.** E-mails: guoyunlong@iccas.ac.cn; liuyq@iccas.ac.cn

^†^Equally contributed to this work

**Contents**

**1. General procedures and experimental details**

**2. Synthetic procedures and characterization**

**3. Fabrication and characterization of OFETs**

**4. Complementary data**

**5. References**

**1. General procedures and experimental details**

Nuclear magnetic resonance (NMR) spectra were recorded on a Bruker DMX-300 (300 MHz) spectrometer. ^1^HNMR chemical shifts were referenced to internal tetramethylsilane (TMS, 0 ppm). High-resolution matrix assisted laser desorption/ionization time-of-flight (HR-MALDI-TOF) mass spectra were collected on an Autoflex III (Bruker Daltonics Inc.) MALDI-TOF spectrometer. Molecular weights were determined by gel permeation chromatography (GPC) at 150 °C on a PL-220 system using 1,2,4-tricholorobenzene as the eluent. TGA measurements were carried out on a PerkinElmer series 7 thermal analysis system under N_2_ at a heating rate of 10 °C min^−1^. UV–vis absorption spectra were measured on polymer solutions in ODCB and polymer films cast onto quartz glass using a Jasco-570 spectrophotometer. For polarized UV−vis absorption, the polymer films were prepared by an on-center or off-center SC method on quartz glass, then were annealed at 120 °C (for PX) or 180 °C (for ODCB). CV was carried out on an electrochemical workstation (CHI660c) using a three-electrode cell. The glassy carbon electrode coated with a thin film layer of polymer was used as the working electrode. Ag/AgCl (Ag in a 0.01 mol/L KCl) electrode was used as the reference electrode. Platinum wire was used as the counter electrode. Anhydrous and N_2_ saturated solution 0.1 M tetrabutylammonium hexylfluorophosphate (*n*-Bu_4_NPF_6_) in acetonitrile was employed as the electrolyte. AFM measurements were carried out on a Nanoscope V instrument. For AFM tests, the polymer films were prepared by an on-center or off-center SC method from PX or ODCB solution on glass substrates, then were annealed at 120 °C (for PX) or 180 °C (for ODCB). In the off-center SC process, the substrates were located 20 mm away from the rotational axis of the spin coater. 2D-GIWAXS was performed on 1W1A Station of Beijing Synchrotron Radiation Facility (λ = 1.54 Å). For 2D-GIWAXS characterizations, the polymer films were prepared by an on-center or off-center SC method from PX or ODCB solution on glass substrates, then were annealed at 120 °C (for PX) or 180 °C (for ODCB). In the off-center SC process, the substrates were located 20 mm away from the rotational axis of the spin coater.

**2. Synthetic procedures and characterization**

Reagents: All starting reagents were purchased from Aldrich, Acros, Alfar Aesar or J&K and used directly without further purification. Compound **1** and **3** were synthesized according to the literature. Synthetic route to **PITTI-BT** was listed as follow:

**6,6'-(thieno[3,2-b]thiophene-2,5-diyl)bis(1-(4-decyltetradecyl)-1H-pyrrolo[2,3-b]pyridine-2,3-dione) (2):** A Schlenk flask was charged with **1** (1.7 g, 3.02 mmol), 2,5-bis(trimethylstannyl)thieno[3,2-b]thiophene (0.586 g, 1.26 mmol) and catalytic amount of Pd_2_(dba)_3_ (115.1 mg, 0.126 mmol), P(*o*-tol)_3_ (304.4 mg, 1.0 mmol) under argon. 20 mL of degassed toluene was added. The mixture was stirred for 24 h at 110 ℃ under argon. Then the solution was cooled to room temperature, the solvent was removed under reduced pressure and the crude product was purified by silica gel chromatography with eluent (PE: CH_2_Cl_2_ = 1:1) to give **2** as a solid (1.2 g, 86.3 %).

^1^H NMR (300 MHz, CDCl_3_, δ): 7.97 (s, 2H), 7.85 (d, *J* = 7.8 Hz, 2H), 7.43 (d, *J* = 7.8 Hz, 2H), 3.89 (t, *J* = 6.9 Hz, 4H), 1.82 (m, 4H), 1.22 (m, 78H), 0.85 (m, 12H). ^13^C NMR (75 MHz, CDCl_3_, δ): 180.6, 164.3, 158.9, 157.6, 148.2, 144.1, 133.3, 120.2, 113.8, 110.4, 39.9, 37.0, 33.5, 31.9, 30.7, 30.2, 29.8, 29.7, 29.4, 26.7, 24.5, 22.7, 14.1. HR-MALDI-TOF: [M+H]^+^ calcd for C_68_H_105_N_4_O_4_S_2_: 1105.75773, found: 1105.75792.

**ITTI-2Br:** In a mixed solvent of acetic acid (30 mL) and chlorobenzene (30 mL), **3** (0.92 g, 1.63 mmol), **2** (0.60 g, 0.54 mmol), tosylic acid (28 mg) were added. The mixture was stirred at 120 ℃ for 48 h under argon. After cooling to room temperature, water and dichloromethane were added. The organic extract was washed with water and then dried over with Na_2_SO_4_. After removal of the solvent under reduced pressure, the crude product was purified by silica gel chromatography with eluent (PE: CH_2_Cl_2_ = 2: 1) to give **ITTI-2Br** as a solid (0.70 g, 58.8 %).

^1^H NMR (300 MHz, CDCl_3_, δ): 9.25 (d, *J* = 8.1 Hz, 2H), 8.94 (d, *J* = 8.7 Hz, 2H), 7.54 (s, 2H), 7.07 (m, 4H), 3.89 (m, 8H), 1.81 (m, 4H), 1.65 (m, 4H), 1.30 – 1.00 (m, 156H), 0.85 (m, 24H). ^13^C NMR (75 MHz, CDCl_3_, δ): 167.3, 167.0, 156.9, 151.5, 148.0, 144.6, 142.8, 141.4, 137.1, 131.9, 131.8, 131.1, 129.9, 126.5, 125.1, 122.9, 118.1, 114.3, 114.1, 114.0, 111.9, 42.7, 39.9, 37.3, 33.7, 33.6, 32.0, 32.0, 31.1, 30.8, 30.4, 30.3, 30.0, 29.9, 29.8, 29.8, 29.5, 29.4, 26.8, 26.7, 26.3, 24.8, 22.8, 14.2. HR-MALDI-TOF: [M+H]^+^ calcd for C_132_H_207_Br_2_F_2_N_6_O_4_S_2_: 2203.39682, found: 2203.39987.

**PITTI-BT:** **ITTI-2Br** (100.0 mg, 0.0454 mmol), 5,5'-bis(trimethylstannyl)-2,2'-bithiophene (22.3 mg, 0.0454 mmol), Pd_2_(dba)_3_ (1.3 mg), P(*o*-tol)_3_ (3.5 mg), and chlorobenzene (4 mL) were added to a Schlenk tube. The tube was charged with argon through a freeze-pump-thaw cycle for three times. The mixture was stirred for 36 h at 120 °C, cooled down to room temperature and poured into methanol (100 mL) and stirred for 3 h. The precipitated product was filtered and purified via Soxhlet extraction with methanol (10 h), acetone (10 h), hexane (10 h), and was finally collected with chloroform. The chloroform fraction was concentrated by evaporation and precipitated into methanol (100 mL) and filtered off to afford the target polymer (95 mg, 94.8 %). GPC: *M*n = 18.3 kDa, *M*w = 69.5 kDa, PDI = 3.80. Anal. calcd for C_140_H_210_F_2_N_6_O_4_S_4_: C 76.17, H 9.59, N 3.81; found: C 75.87, H 9.48, N 3.78.

**3. Fabrication and characterization of OFETs**

The OFET devices were fabricated with a top-gate bottom-contact (TGBC) configuration on 0.5 cm × 0.5 cm Corning glass substrates. The substrates were cleaned by deionized water, acetone and ethanol under ultrasonication for 10 min. The prepatterned Au/Cr (25 nm/5 nm) layer was prepared as source-drain electrodes by photolithography technique. The channel width and length of all FET devices were 1400 and 40 μm, respectively. The substrates were dried by high-purity nitrogen before device fabrication. The semiconducting material of PITTI-BT was dissolved in PX or ODCB with a concentration of 8 mg/mL. Before the deposition of semiconductor layer, the PX or ODCB solution was preheated at 40 ℃ or 60 ℃, respectively. The glass substrates for devices prepared from PX or ODCB were preheated at 40 ℃ or 60 ℃, respectively. In a nitrogen glovebox, the hot polymer solutions were deposited onto the hot substrates by conventional on-center SC at 2000 rpm. The off-center spin-coated films were prepared using the same experimental conditions as those used for the on-center SC except for the position of the substrates, which were placed 20 mm away from the rotational axis of the spin coater. Then the films were further annealed at 120 °C (for PX) or 180 °C (for ODCB) for 10 min to remove the corresponding solvents. The average thickness of on-center spin-coated films was 20.1 nm. The average thickness of off-center spin-coated films was 10.2 nm. Next, polymethyl methacrylate (PMMA, *M*_W_ = 996 kDa) was spin-coated as dielectric layer onto the surface of the polymer film (PMMA thickness ~700 nm, capacitance ~3.41 nF/cm^2^) at 2000 rpm. PMMA was dissolved in *n*-butyl acetate with a concentration of 60 mg/mL. The samples were thermally baked at 90 °C for 50 min in glove box. Finally, the devices were completed after the evaporation of aluminum as gate electrode (thickness ~90 nm) on PMMA through designed shadow masks. All the devices were determined under the ambient conditions using a Keithley 4200 SCS semiconductor parameter analyzer. The field-effect mobility in saturation region (μ*_sat_*) is calculated according to the equation:

I_DS_ = (W/2L) C_i_ μ*_sat_* (V_GS_ – V_th_)^2^

Where I_DS_ is the drain current; W and L are the semiconductor channel width and length, respectively; C_i_ is the capacitance per unit area of the gate dielectric layer; and V_GS_ and V_th_ are the gate voltage and threshold voltage, respectively.

**4. Complementary data**

**Supplementary Table 1.** Reported ambipolar or n-type polymers processed from nonchlorinated solvents according to literature.

| Polymer | μ_h, max_  [cm^2^V^–1^s^–1^] ^a^ | | μ_e, max_  [cm^2^V^–1^s^–1^] ^a^ | Solvent | Reference |
| --- | --- | --- | --- | --- | --- |
| PDPP2DT-F2T2 | 1.28 | 0.39 | | 1,2,4-trimethyl  -benzene | 3 |
| PDPPF-DFT | 0.26 | 0.12 | | hexane | 4 |
| PDBTz | ‒ | 0.31 | | p-xylene | 5 |
| PDBPyBTz | ‒ | 0.03 | | p-xylene | 6 |
| PDPP4Tz | ‒ | 0.067 | | p-xylene | 7 |
| P(NDI2OD-T2) | ‒ | 0.574 | | mesitylene/  acetophenone | 8 |
| PITTI-BT | 3.06 | 2.81 | | p-xylene | This work |

**Supplementary Table 2.** Number average molecular weights, polydispersity index (PDI), absorption maxima, bandgaps, HOMO and LUMO energy levels of PITTI-BT.

| Polymer | M_n_ [kDa]/PDI | λ_max_  [nm] | E_g_^opt a^  [eV] | HOMO^b^  [eV] | LUMO  [eV] |
| --- | --- | --- | --- | --- | --- |
| PITTI-BT | 18.3/3.80 | 749^c^/752^d^ | 1.52 | –5.71 | –4.19 |

^a^Determined from the onset of thin-film absorption; ^b^Determined from the onset of oxidation and reduction potentials of cyclic voltammetry; ^c^Absorption maximum in solution; ^d^Absorption maximum in film.

**Supplementary Table 3.** Crystallographic information of polymer films prepared from PX

|  | **Crystallographic parameters** | | | |
| --- | --- | --- | --- | --- |
| **On-center** |  | lamella  packing  (100) | *q* (Å^‒1^) | 0.257 |
|  |  |  | *d*-spacing (Å) | 24.4 |
|  |  | *π*–*π* stacking  (010) | *q* (Å^‒1^) | 1.801 |
|  |  |  | *d*-spacing (Å) | 3.49 |
| **Off-center** | **Parallel** | lamella  packing  (100) | *q* (Å^‒1^) | 0.254 |
|  |  |  | *d*-spacing (Å) | 24.7 |
|  |  | *π*–*π* stacking  (010) | *q* (Å^‒1^) | 1.797 |
|  |  |  | *d*-spacing (Å) | 3.50 |
|  | **Perpendicular** | lamella  packing  (100) | *q* (Å^‒1^) | 0.250 |
|  |  |  | *d*-spacing (Å) | 25.1 |
|  |  | *π*–*π* stacking  (010) | *q* (Å^‒1^) | ‒ |
|  |  |  | *d*-spacing (Å) | ‒ |

**Supplementary Table 4.** Crystallographic information of polymer films prepared from ODCB

|  | **Crystallographic parameters** | | | |
| --- | --- | --- | --- | --- |
| **On-center** |  | lamella  packing  (100) | *q* (Å^‒1^) | 0.261 |
|  |  |  | *d*-spacing (Å) | 24.1 |
|  |  | *π*–*π* stacking  (010) | *q* (Å^‒1^) | 1.804 |
|  |  |  | *d*-spacing (Å) | 3.48 |
| **Off-center** | **Parallel** | lamella  packing  (100) | *q* (Å^‒1^) | 0.256 |
|  |  |  | *d*-spacing (Å) | 24.5 |
|  |  | *π*–*π* stacking  (010) | *q* (Å^‒1^) | 1.796 |
|  |  |  | *d*-spacing (Å) | 3.50 |
|  | **Perpendicular** | lamella  packing  (100) | *q* (Å^‒1^) | 0.252 |
|  |  |  | *d*-spacing (Å) | 24.9 |
|  |  | *π*–*π* stacking  (010) | *q* (Å^‒1^) | ‒ |
|  |  |  | *d*-spacing (Å) | ‒ |

**Supplementary Table 5**. TGBC OFET parameters of PITTI-BT

| Solvent | Method | *μ_h_*^a^  [cm^2^V^–1^s^–1^] | *V*_th_^b^  [V] | *I*_on_/*I*_off_^c^ | *μ*_e_^a^  [cm^2^V^–1^s^–1^] | *V*_th_^b^  [V] | *I*_on_/*I*_off_^c^ |
| --- | --- | --- | --- | --- | --- | --- | --- |
| PX | on center | 1.51 (1.06) | -44 (±4) | 10^3^-10^4^ | 1.31 (0.95) | 71 (±4) | 10^3^-10^4^ |
|  | off center (parallel) | 3.06 (2.31) | –53 (±5) | 10^4^-10^5^ | 2.81 (1.87) | 84 (±4) | 10^3^-10^4^ |
|  | off center (perpendicular) | 0.91 (0.69) | -56 (±5) | 10^3^-10^4^ | 0.80 (0.59) | 78 (±3) | 10^3^-10^4^ |
| ODCB | on center | 2.22 (1.57) | –68 (±4) | 10^3^-10^4^ | 1.97 (1.20) | 66 (±3) | 10^3^-10^4^ |
|  | off center (parallel) | 4.72 (4.14) | –66 (±4) | 10^3^-10^4^ | 4.11 (3.53) | 57 (±3) | 10^3^-10^4^ |
|  | off center (perpendicular) | 1.21 (0.87) | –55 (±5) | 10^3^-10^4^ | 1.03 (0.67) | 58 (±5) | 10^3^-10^4^ |

^a^Maximum mobilities extracted from the transfer curves in the saturation regimes. The average values are listed in parentheses. ^b^Threshold voltage; ^c^On–off current ratio


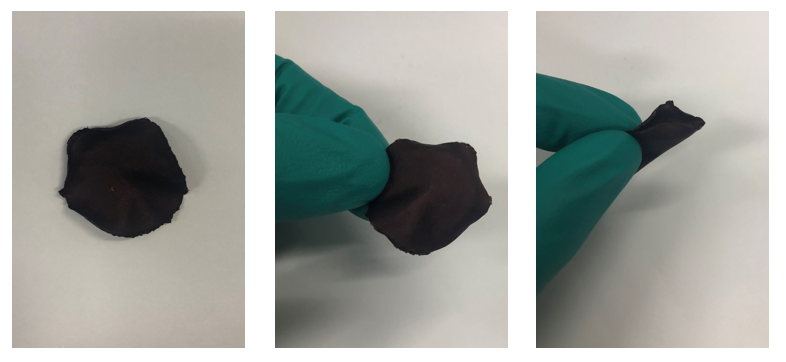


**Supplementary Figure 1.** Photographs for the free-standing films of PITTI-BT.


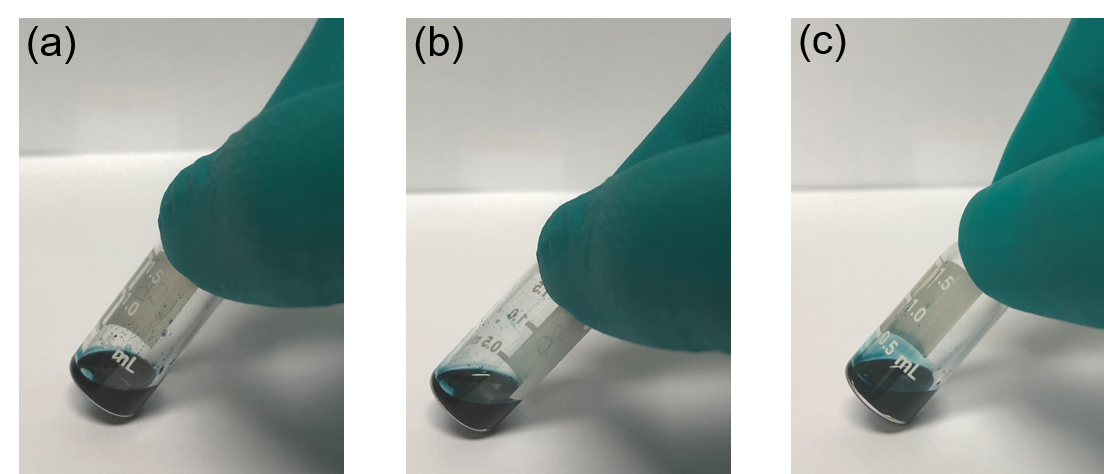


**Supplementary Figure 2.** Solubility tests of PITTI-BT in different solvents such as (a) chlorobenzene, (b) o-dichlorobenzene, and (c) p-xylene. We focused on comparing the fluidity of polymer solutions in different solvents. The solutions were treated under ultrasonication for 10 minutes and then heated at 90 °C for 5 h. The same vials were stored at 25 °C for 12 h.


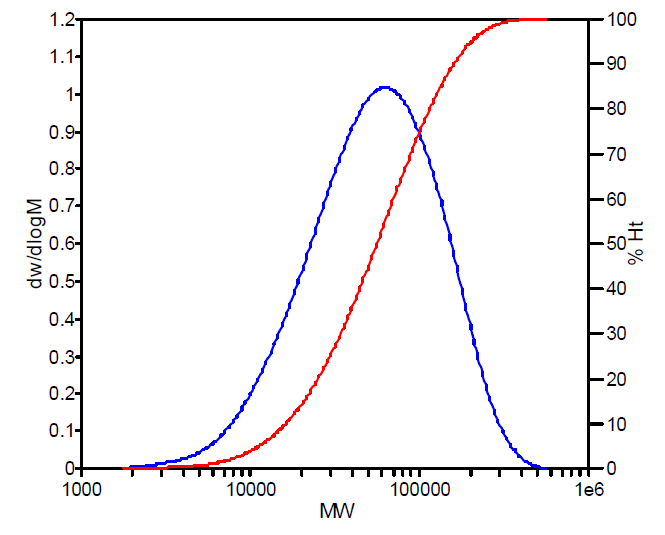


**Supplementary Figure 3.** Gel permeation chromatography (GPC) trace of PITTI-BT.

**
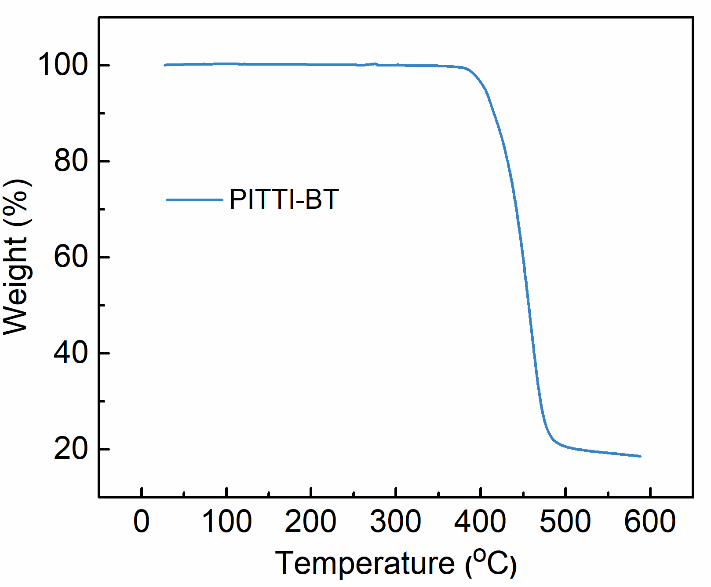
**

**Supplementary Figure 4.** Thermal gravity analysis (TGA) of PITTI-BT.


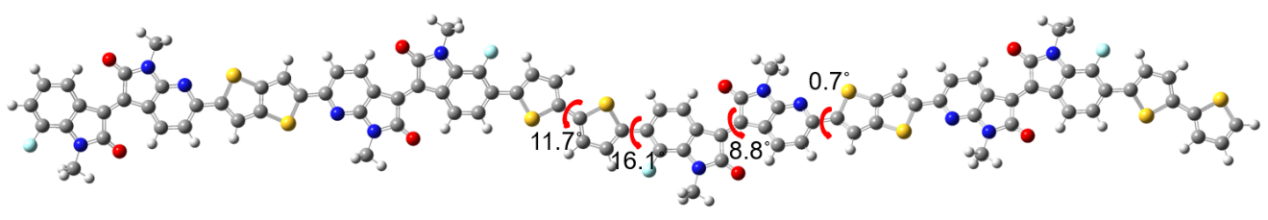


**Supplementary Figure 5.** DFT optimized geometry of a PITTI-BT dimer from the top view. Calculated at DFT B3LYP/6-31G(d) level. The planarity data was listed. DFT calculations were performed on a methyl-substituted dimer of PITTI-BT.


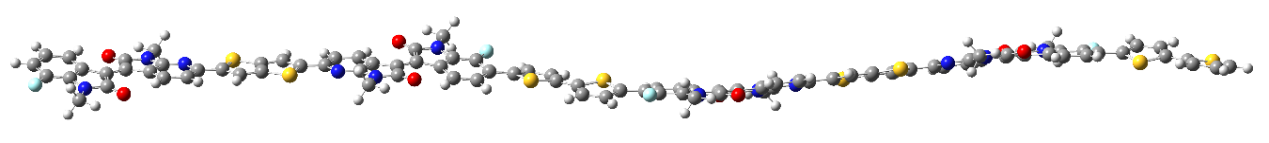


**Supplementary Figure 6.** DFT optimized geometry of a PITTI-BT dimer from the side view. Calculated at DFT B3LYP/6-31G(d) level.

**
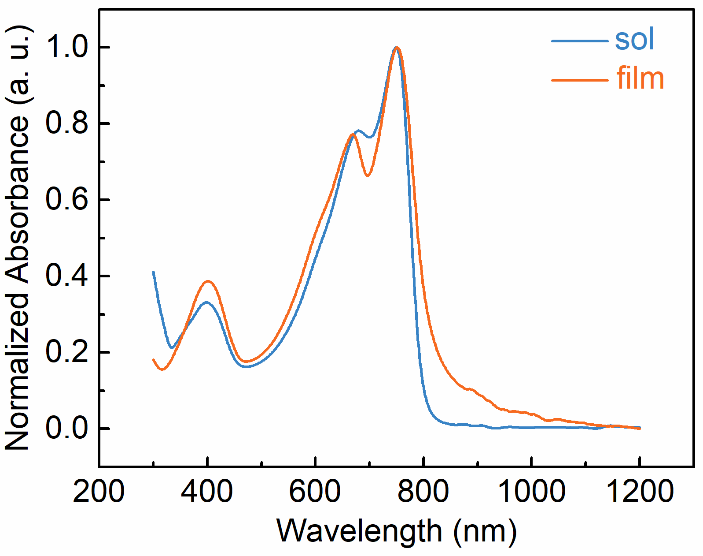
**

**Supplementary Figure 7.** UV−vis absorption spectra of PITTI-BT in chloroform solution and in thin film.


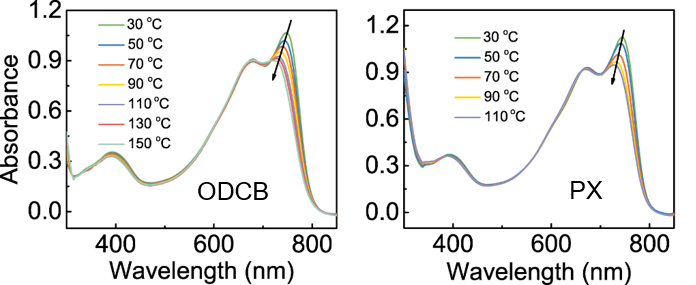


**Supplementary Figure 8.** Temperature-dependent UV−vis absorption spectra of PITTI-BT in ODCB or PX solution.


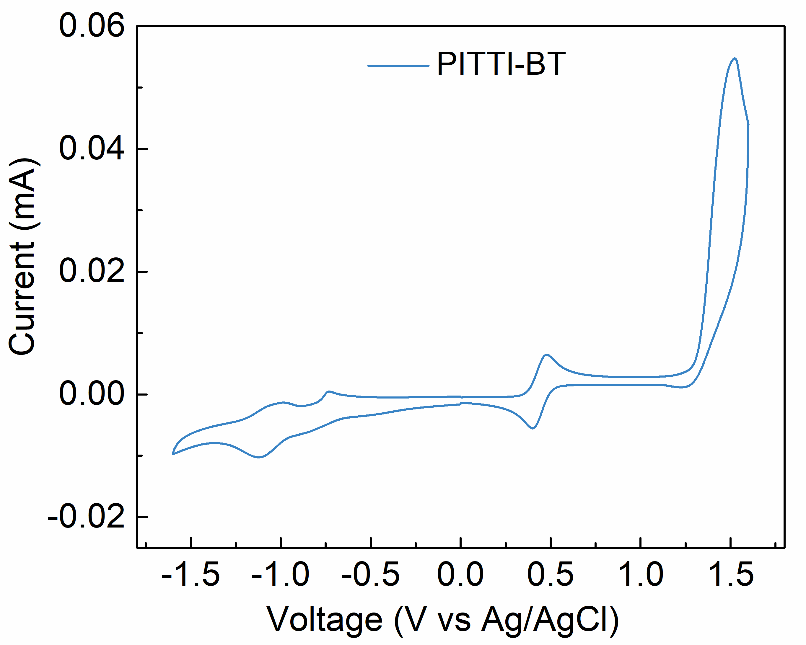


**Supplementary Figure 9.** Cyclic voltammetry of PITTI-BT. Ferrocene was used an internal standard.

**
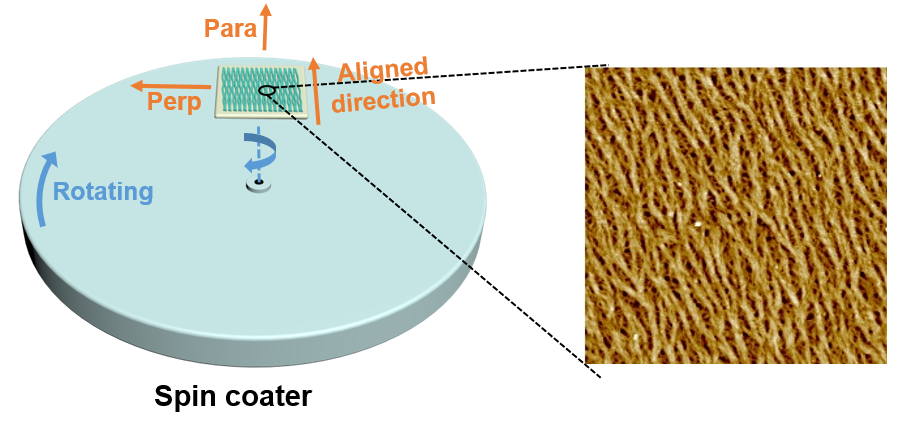
**

**Supplementary Figure 10.** Schematic illustration of an off-center SC method. The aligned direction of polymer film is along the radial direction.

**
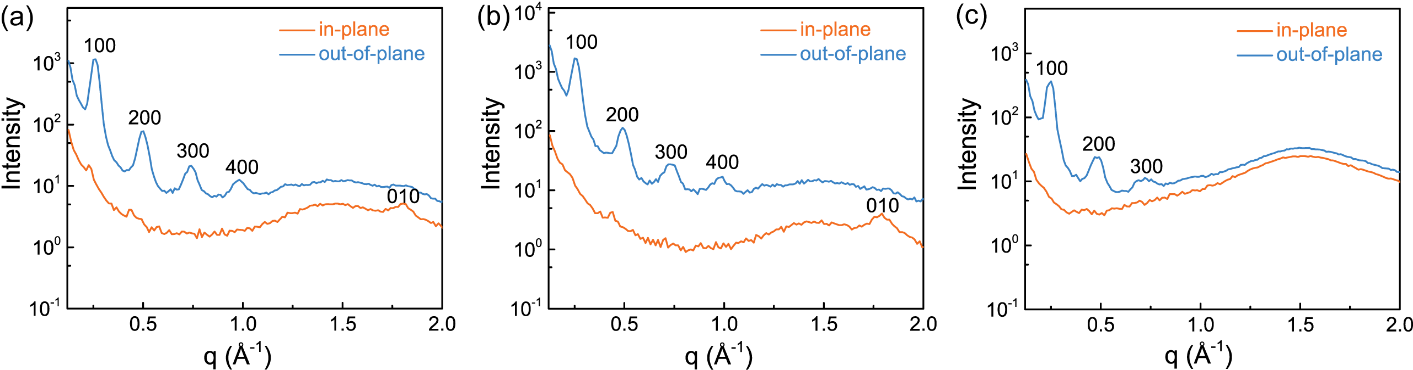
**

**Supplementary Figure 11.** The in-plane and out-of-plane profiles of 2D-GIWAXS of polymer films prepared from PX solution by (a) on-center SC, (b) off-center SC (parallel) and (c) off-center SC (perpendicular) methods.


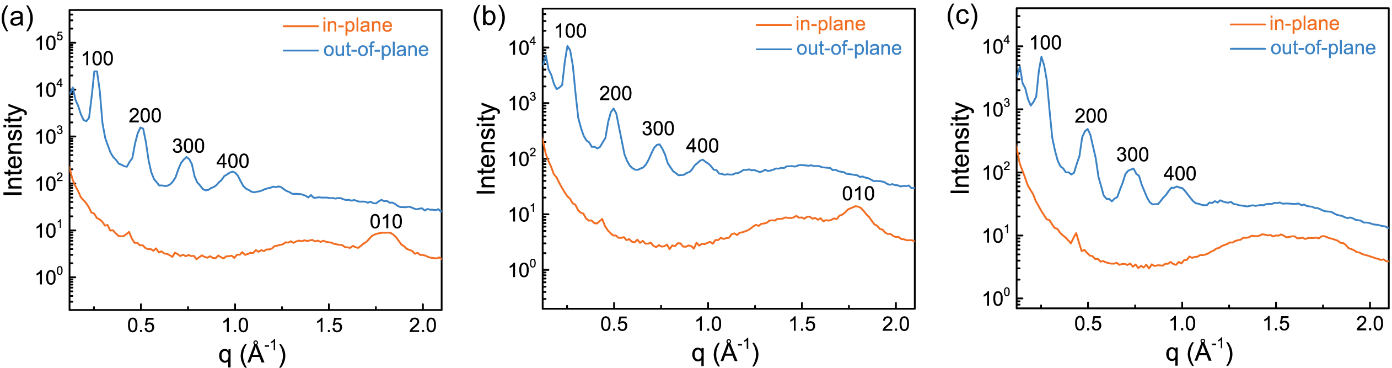


**Supplementary Figure 12.** The in-plane and out-of-plane profiles of 2D-GIWAXS of polymer films prepared from ODCB solution by (a) on-center SC, (b) off-center SC (parallel) and (c) off-center SC (perpendicular) methods.


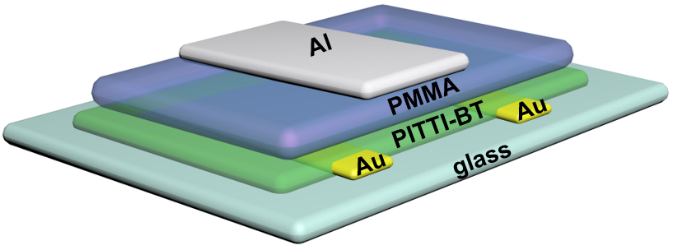


**Supplementary Figure 13.** Schematic device configuration of TGBC OFETs.


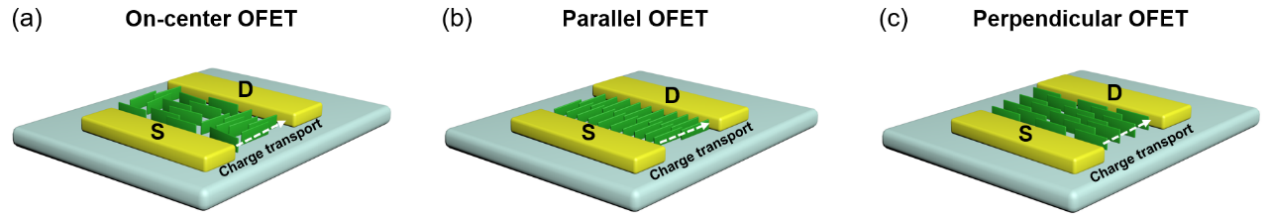


**Supplementary Figure 14.** (a) The devices fabricated by an on-center SC method. (b,c) The devices fabricated by an off-center SC method. The transistor channels were (b) parallel or (c) perpendicular to the film aligned direction. For visualization, the PMMA layers and gates were not shown.

**
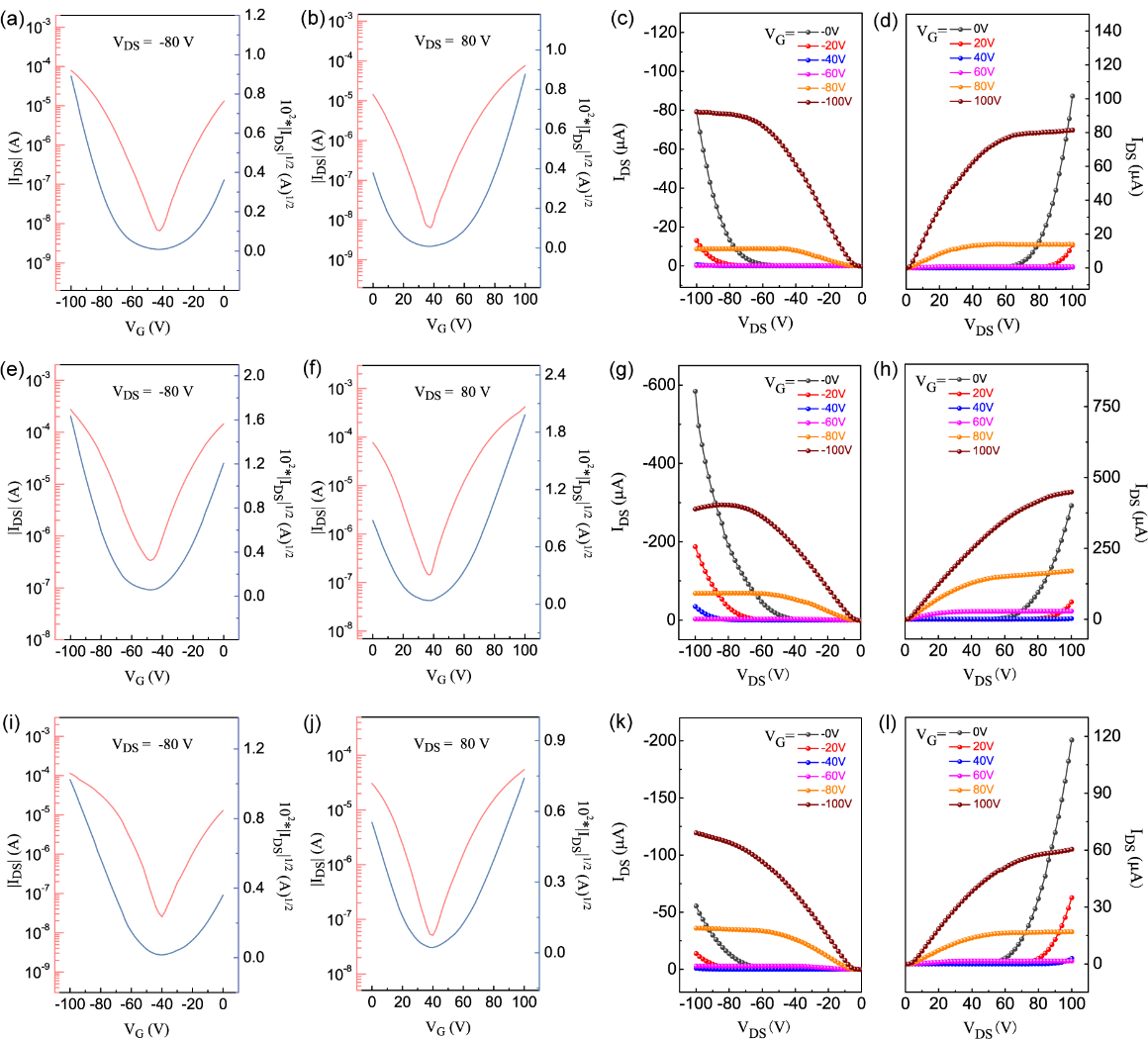
**

**Supplementary Figure 15.** Transfer and output characteristics of OFETs prepared by (a–d) on-center and (e–l) off-center SC methods from ODCB solution. The transistor channels are (e–h) parallel or (i–l) perpendicular to the film aligned direction.


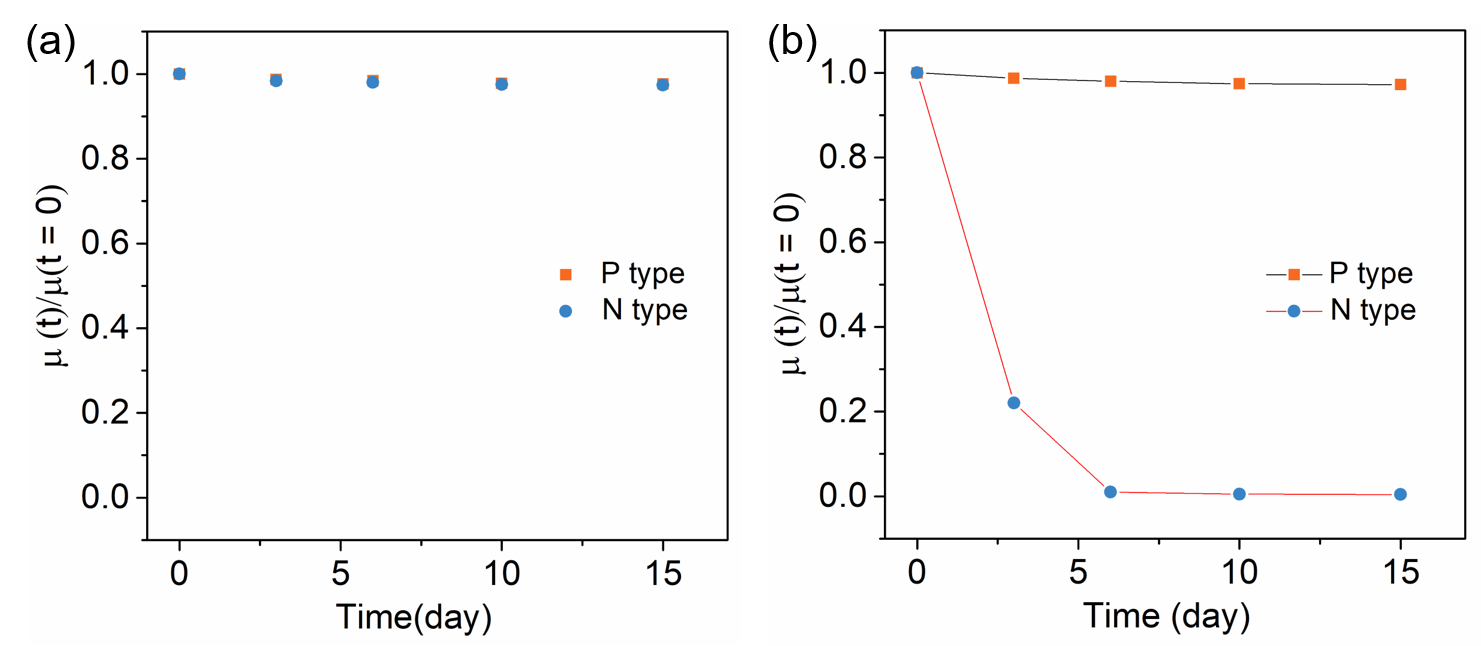


**Supplementary Figure 16.** Time-dependent variations of the mobilities of PITTI-BT-based OFETs while stored (a) in a nitrogen glovebox or (b) in air (RH = 30~40%). The stability was tested based on top-gate bottom-contact (TGBC) OFETs fabricated by an off-center SC method from ODCB solution.


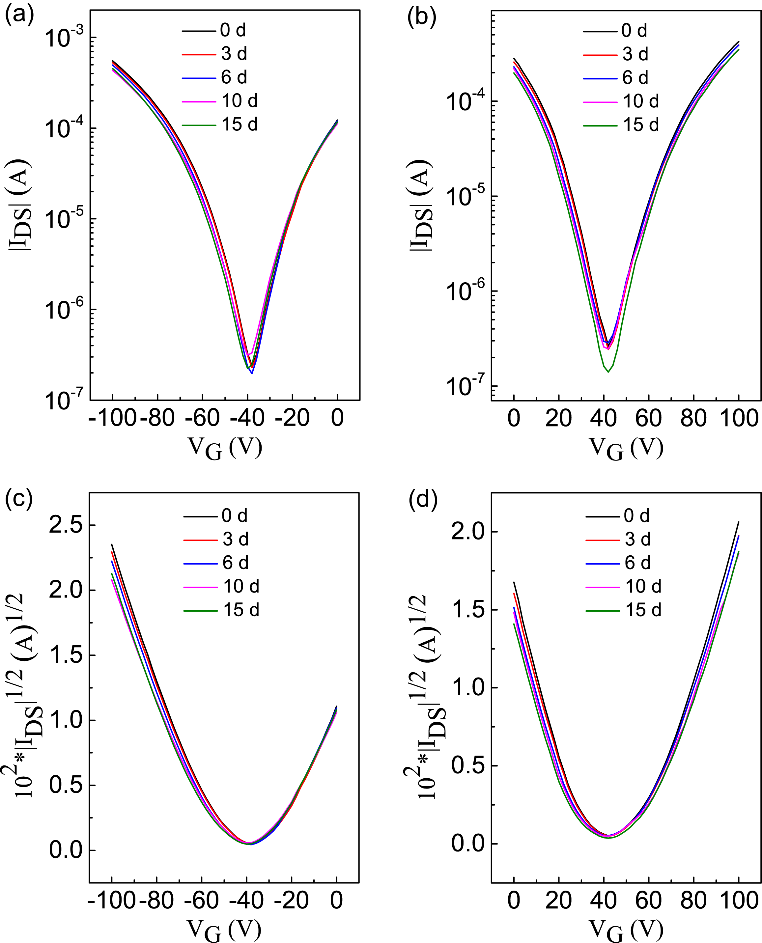


**Supplementary Figure 17.** (a, c) P-type (V_DS_ = −80 V) and (b,d) n-type (V_DS_ = 80 V) transfer characteristics of OFETs tested immediately after device fabrication (fresh) and after the devices were stored in a nitrogen glovebox for several days (corresponding to Supplementary Figure 16a). The hole and electron mobilities of fresh devices were 4.35 and 3.85 cm^2^ V^−1^ s^−1^, respectively.


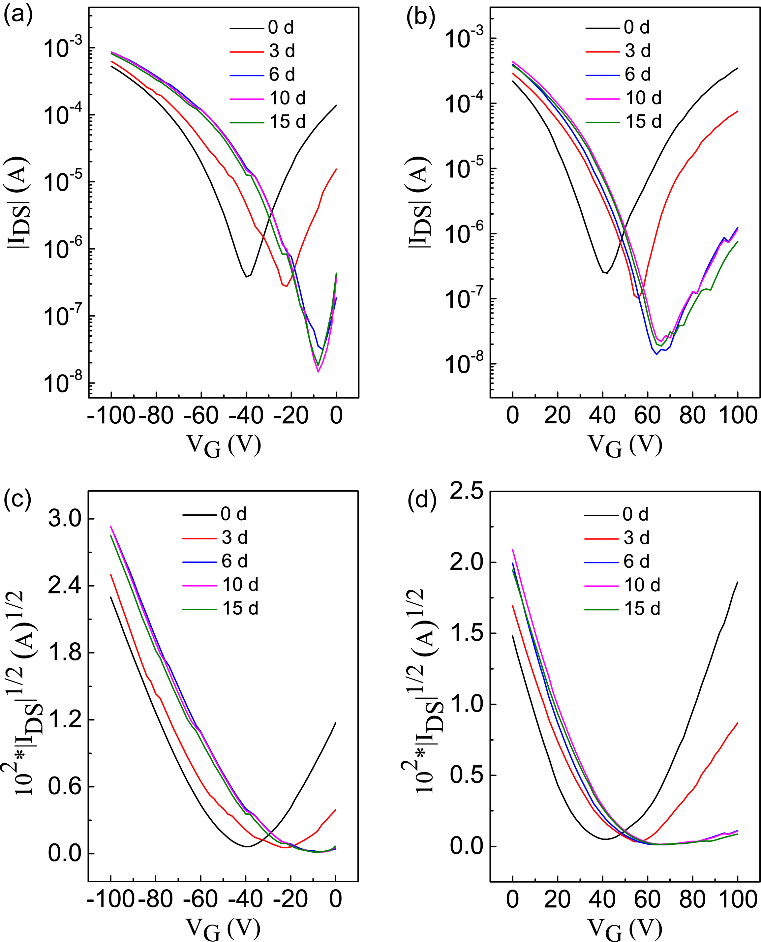


**Supplementary Figure 18.** (a,c) P-type (V_DS_ = −80 V) and (b,d) n-type (V_DS_ = 80 V) transfer characteristics of OFETs tested immediately after device fabrication (fresh) and after the devices were stored in air for several days (RH = 30~40%) (corresponding to Supplementary Figure 16b). The hole and electron mobilities of fresh devices were 4.20 and 3.47 cm^2^ V^−1^ s^−1^, respectively.

**
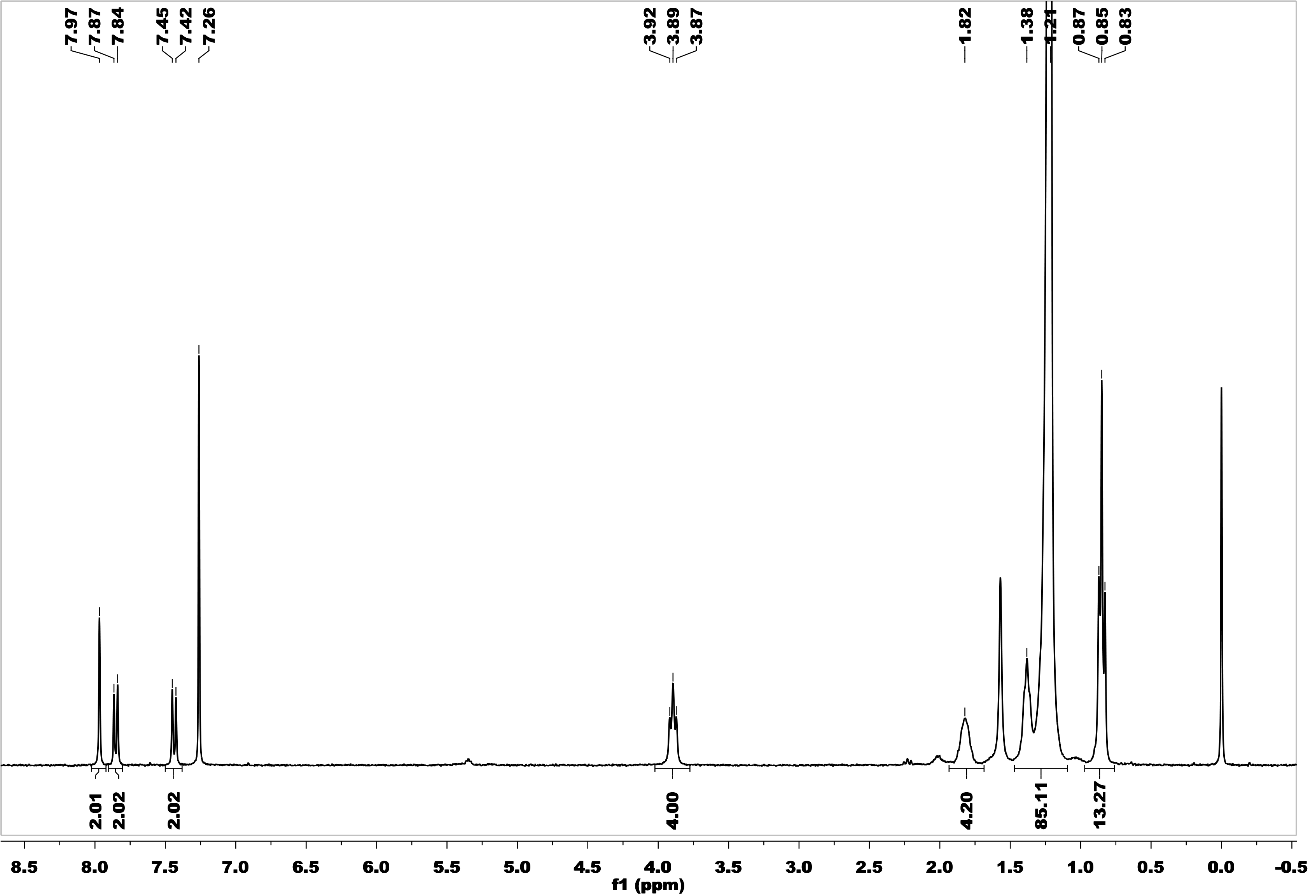
**

**Supplementary Figure 19.** The ^1^H NMR spectrum of **Compound 2**.


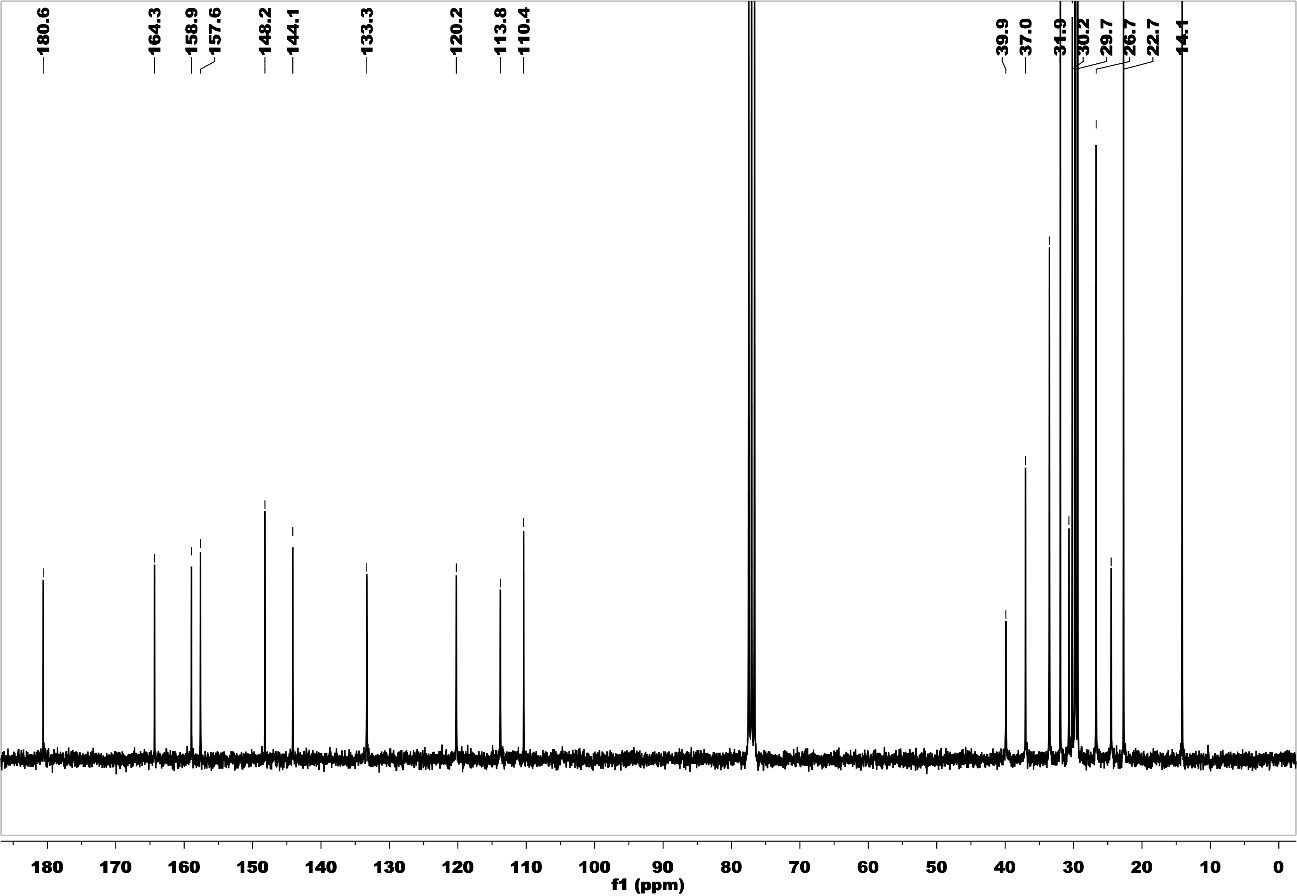


**Supplementary Figure 20.** The ^13^C NMR spectrum of **Compound 2**.


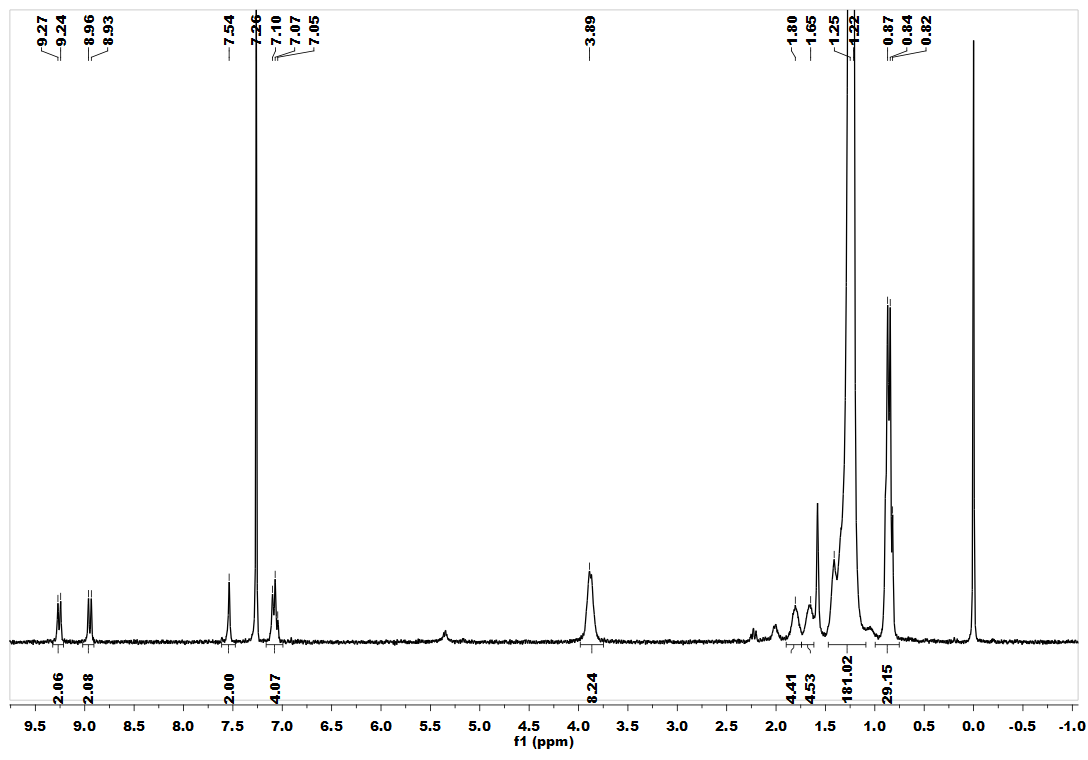


**Supplementary Figure 21.** The ^1^H NMR spectrum of **ITTI-2Br**.


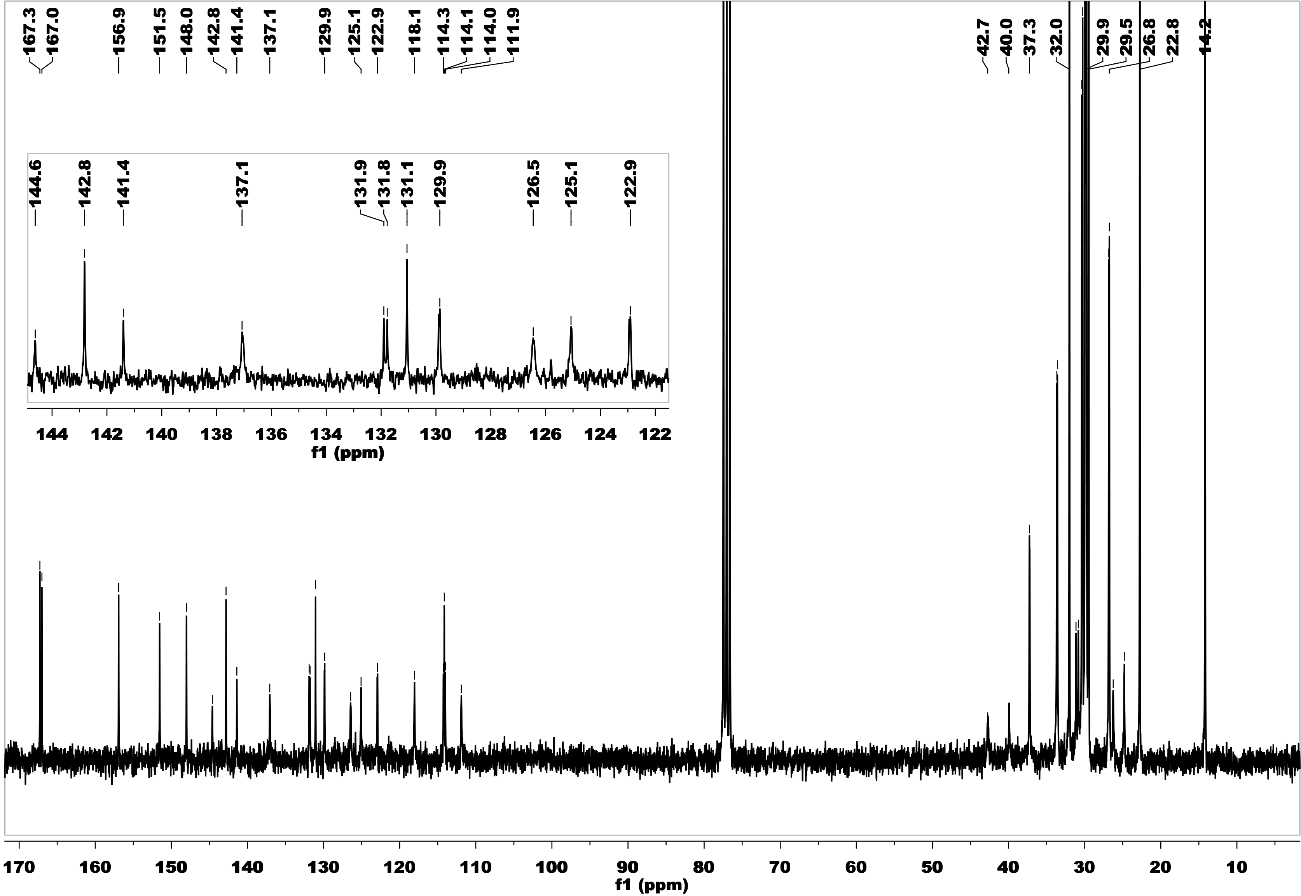


**Supplementary Figure 22.** The ^13^C NMR spectrum of **ITTI-2Br** (inset: amplified ^13^C NMR spectrum (122–144 ppm)).

**5. References**

1. Huang J, Mao Z and Chen Z *et al.* Diazaisoindigo-based polymers with high-performance charge-transport properties: From computational screening to experimental characterization. *Chem Mater* 2016; **28**: 2209–18.

2. Yang J, Zhao Z and Geng H *et al.* Isoindigo‐based polymers with small effective masses for high‐mobility ambipolar field‐effect transistors. *Adv Mater* 2017; **29**: 1702115.

3. Lee M, Kim MJ and Ro S *et al.* A nonchlorinated solvent-processable fluorinated planar conjugated polymer for flexible field-effect transistors. *ACS Appl Mater Interfaces* 2017; **9**: 28817–27.

4. Sonar P, Chang J and Kim JH *et al.* High-mobility ambipolar organic thin-film transistor processed from a nonchlorinated solvent. *ACS Appl Mater Interfaces* 2016; **8**: 24325–30.

5. Fu B, Wang C-Y and Rose BD *et al.* Molecular engineering of nonhalogenated solution-processable bithiazole-based electron-transport polymeric semiconductors. *Chem Mater* 2015; **27**: 2928–37.

6. Buckley C, Thomas S and McBride M *et al.* Synergistic use of bithiazole and pyridinyl substitution for effective electron transport polymer materials. *Chem Mater* 2019; **31**: 3957–66.

7. Yuan Z, Fu B and Thomas S *et al.* Unipolar electron transport polymers: A thiazole based all-electron acceptor approach. *Chem Mater* 2016; **28**: 6045–9.

8. Opoku H, Nketia-Yawson B and Shin E-S *et al.* Organic field-effect transistors processed by an environmentally friendly non-halogenated solvent blend. *J Mater Chem C* 2018; **6**: 661–7.
